# Leukopenia: Its Relation to Neuralgia

**Published:** 1916-05

**Authors:** 


**Leukopenia: Its Relation to Neuralgia.** Joseph H. Catton of San Francisco, Calif. State Jour. Mch., presents a bibliography and concludes as follows:

(1) Neuralgia may occur:

a. In exogenous infectious intoxications: malaria, influenza, syphilis, and less frequently typhoid, tuberculosis, smallpox and tonsilitis.

b. Exogenous non-infectious intoxications: Poisoning with lead, arsenic, mercury, copper, nicotine, hydrogen suphide, illuminating gas, benzene and iodine; or as an abstinence symptom in morphinism and cocainism.

c. Dyscrasic intoxication in blood disease: the severe anemias, pernicious anemia, chlorosis, leukemias, hemophilia and polycythemia.

d. Following exposure to cold.

e. In certain ductless gland disturbances.

f. In debility and malnutrition.

(2) A relative or absolute reduction in the number of granular leukocytes tends to be characteristic of each of the above conditions.

Conclusion—After a study of the conditions etiological in neuralgia and of the leukocyte pictures present in these conditions, the suggestion is offered that there is a definite relation between neuralgia on the one hand and a disturbance in the normal polynuclear-lymphocyte balance,—a tendency toward decrease in the number of granular cells and increase in the number of hyaline ones.

